# Oxidation behaviour of EUROFER-97 under simulated water-cooled lithium lead breeder blanket conditions

**DOI:** 10.1038/s41529-025-00600-y

**Published:** 2025-05-13

**Authors:** Liberato Volpe, Dora Capone, Peter Andresen, Eric Prestat, Fabio Scenini

**Affiliations:** 1 Materials Division, United Kingdom Atomic Energy Authority, Culham Campus, Abingdon, OX14 3DB UK; 2 Department of Materials, Henry Royce Institute, The University of Manchester Oxford Road, Manchester, M13 9PL UK; 3 Paul Scherrer Institute (PSI), Center for Nuclear Engineering and Sciences (NES), Laboratory for Nuclear Materials, 5232 Villigen PSI, Switzerland; 4 Andresen Consulting, 12204 Wildwood Park Place, Bakersfield, CA 93311 USA

**Keywords:** Metals and alloys, Corrosion

## Abstract

The effect of water chemistry, surface condition, alkalizing agent (LiOH *vs*. KOH), and Zn addition was investigated at 300 °C on the oxidation behaviour of reduced activation ferritic martensitic (RAF/M) EUROFER-97. EUROFER-97 is the proposed material for the water-cooled lithium lead breeder blanket (WCLL-BB) section of DEMO, but its behaviour under elevated temperature hydrogenated water has never been investigated. Advanced material characterization showed that, despite its relatively low chromium content, EUROFER-97 exhibits high corrosion resistance. This is because EUROFER-97 is protected by an inner polycrystalline FeCr_2_O_4_ layer, formed regardless of the water chemistry and surface preparation investigated. The outer non-protective oxide consists of Fe_3_O_4_ crystallites, which were refined when KOH was used. When injected, Zn was observed only on top of the outer crystallites without diffusing into the inner oxide layer. These findings demonstrate the excellent oxidation behaviour of EUROFER-97 in the proposed water chemistry, highlighting its suitability for the WCLL-BB section.

## Introduction

Breeder blanket section in a tokamak fusion reactor, such as ITER and DEMO (DEMOnstration power plant) plays an important role for the reactor^1–4^, as it breeds tritium from ^6^Li, acts as first shielding material and it absorbs energy from the high energy neutrons (up to 14 MeV) occurring from the deuterium – tritium (D-T) reaction^5^. The main structural material for the breeder blanket is a RAF/M steel in which Mo and Ni have been replaced with W, V and Ta^5^ to minimise transmutation occurring with the interaction with the high-energy neutrons. The RAF/M EUROFER-97 is the European flagship RAF/M steels, and for the breeder blanket water coolant (WCLL-BB) configuration, it will face environmental conditions similar to those used in a primary circuit of a pressurised water reactor (PWR). EUROFER-97 is not classified as stainless steel due to its 9% chromium concentration, which is lower than the 10.5% required for standard stainless steel and less than the 12% conventionally accepted as the minimum passivity^6^. However, there is little characterization of oxide formation for medium Cr alloys (9–12 wt.%) and there is no water chemistry specification for the newly WCLL-BB component for fusion. Generally the water chemistry in a primary circuit of a PWR is accurately set to avoid general corrosion and environmentally assisted cracking (EAC)^7–10^: Hydrogen (H_2_) is added in the range of 2–4 ppm to lower the electrochemical corrosion potential (*EcP*) to -750 mV *vs*. SHE (standard hydrogen electrode) to suppress the formation of oxidising radiolysis products (e.g., O_2_, H_2_O_2_), boron (B) is added in the range of 1000 ppm as neutron moderator, and lithium hydroxide (LiOH) is added in the range of 2–4 ppm to maintain the *pH*_325 °C_ between 6.9 and 7.4 and improve the passivity of the exposed metals^11–13^. It is also worth mentioning the interest to use for the PWR of KOH as alkalising agent, due to concerns arisen over the supply chain security of the ^7^Li^14^, thus providing security in materials supply and potential cost reduction^15^. Currently, the use of KOH in PWRs is being mainly investigating to assess the EAC behaviour^16–20^ of stainless steels and Ni-base alloy, however, very little work has been performed on oxide formation and cation deposition^21^ and none has been performed on materials of interest for fusion (e.g., reduced activation ferritic/martensitic steels and CuCrZr alloys).

Co released from stainless steels (SSs), present outside the BB zone^22^, will be activated and incorporated into oxides throughout the system, undermining the use of the reduced activation composition of EUROFER-97. It has been shown from previous experience from boiling water reactors (BWRs) that the presence of Zn cations into the oxide of SSs induces the formation of a more protective Zn-based spinel oxide film^23,24^ that displaces and mitigates the build-up of ^60^Co [14,15]. In fact, Zn, when added in the feedwater (≥ 5 ppb)^25–28^ competes as Zn^2+^ with Co^2+^ in the tetrahedral interstitial sites of the outer spinel oxides, hindering Co incorporation in the oxide^26,27^, and reducing the gamma radiation^29^ associated with the neutron activated ^60^Co^30^. However, to the knowledge of the authors, not work has been performed on the RAF/M EUROFER-97. Moreover, the overall corrosion^31–33^ and EAC^32–34^ behaviour under PWR-like environmental conditions can be also affected (and therefore mitigated) by the surface preparation, and two different surface preparations were used to assess if the same impact on oxide evolution observed in other alloys also applies to EUROFER-97.

The aim of this work was to understand the oxide formation to extract the mechanistic understanding required to develop a suitable water chemistry for the WCLL-BB component to have confidence in the durability and activation of EUROFER-97 for its designed fusion applications. In this work, flat coupons of EUROFER-97 with different surface conditions (OPS (oxide silica polishing suspension) *vs*. P320 ground) were prepared with two different surface finish, The coupons were then exposed to either LiOH, KOH and KOH with 5 ppb Zn to reproduce different potential WCLL-BB water chemistries at *pH*_300°C_ equal to 7.4 to understand the impact of the different water chemistries selected on the oxidation behaviour of EUROFER-97. The morphology of the oxide formed after exposure was then revealed using complementary microstructural techniques, including scanning electron microscopy (SEM) and analytical transmission electron microscopy (ATEM).

## Results

### SEM characterisation after high-temperature water exposure

**Fig. 1:**
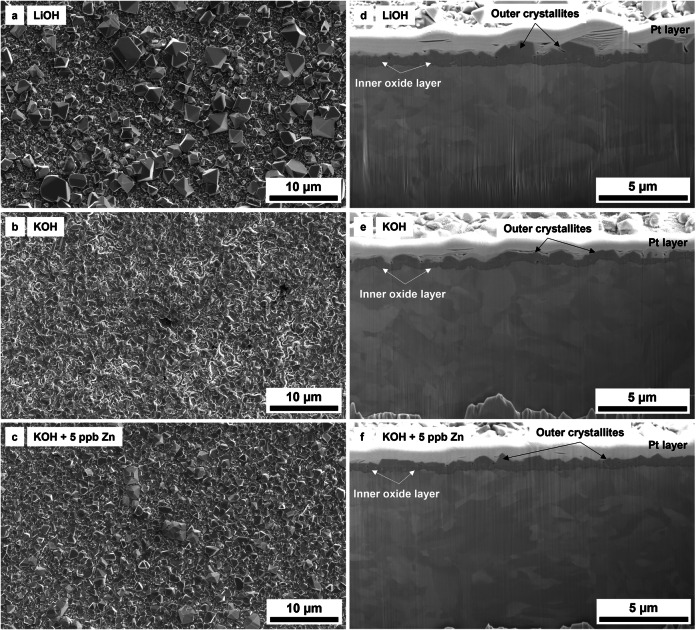
Overview of the surface and FIB cross section of the oxidised EUROFER-97 specimens prepared with an OPS surface. **a**–**c** SEM SE plan views and (**d–f**) FIB cross-sectional views of the OPS polished EUROFER-97 coupons exposed to (**a, d**) LiOH, (**b, e**) KOH and (**c, f**) KOH with 5 ppb Zn.

**Fig. 2:**
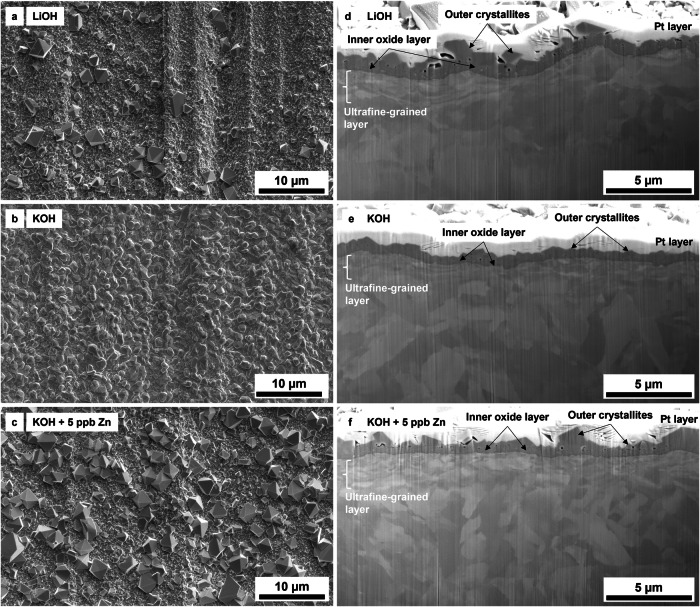
Overview of the surface and FIB cross-section of the oxidised EUROFER-97 specimens prepared with a P320 emery paper. **a**–**c** SEM SE top and (**d–f**) FIB cross-sectional views of P320 ground EUROFER-97 coupons exposed to (**a, d**) LiOH, (**b, e**) KOH and (**c, f**) KOH with 5 ppb Zn.

**Fig. 3:**
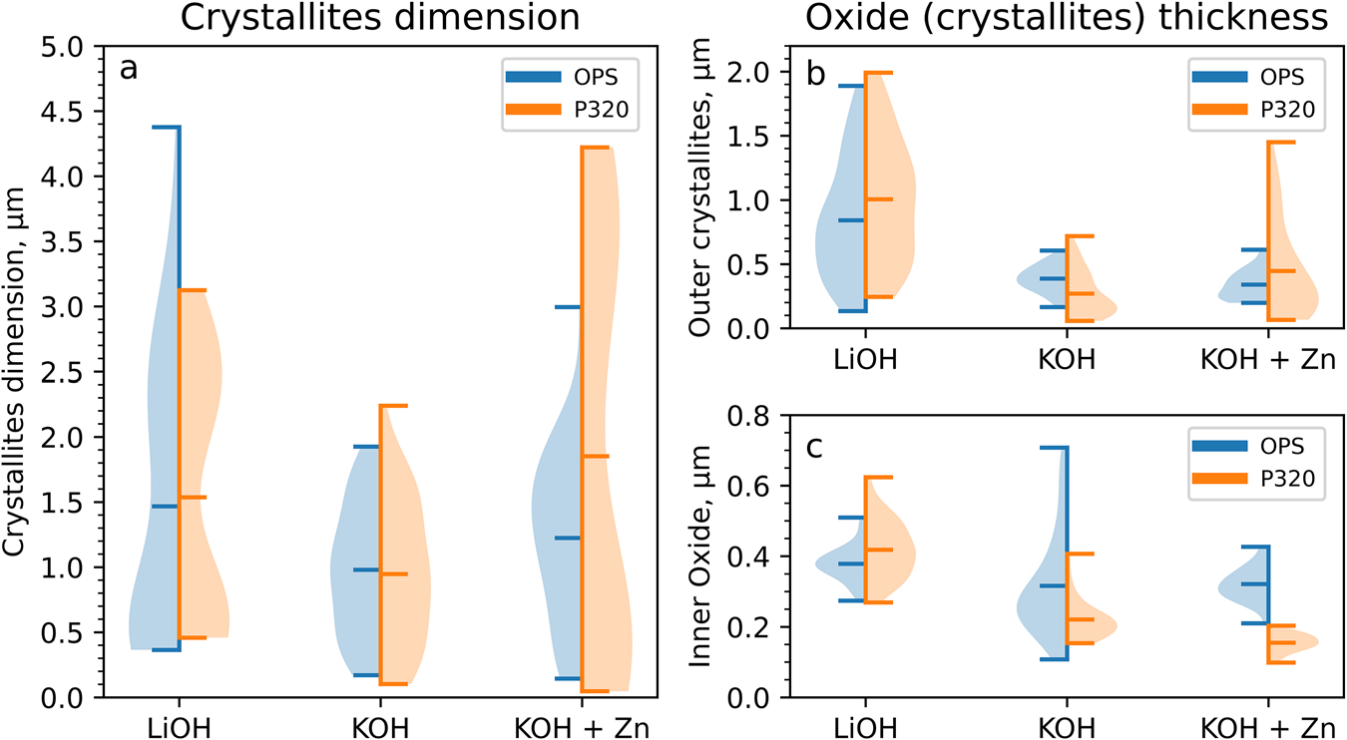
Size and thickness distribution of the outer and inner oxide. **a** Summary of the dimensions of the outer crystallites and (**b**) outer and (**c**) inner oxide thickness for the OPS and P320 prepared EUROFER-97 coupons exposed under LiOH, KOH and KOH with 5 ppb Zn at *pH* = 7.4 at 300 °C.

Representative top and cross-sectional views of the oxidised EUROFER-97 coupons prepared with the OPS and P320 surfaces are shown in Figs. 1 and 2 with the aim of assessing the oxide formed onto the surface after exposure to the simulated WCLL-BB environment. The surface (Fig. 1a, b, c) of the three EUROFER-97 coupons prepared with the OPS finish was covered with a continuous layer of crystallites. A bimodal size distribution of the crystallites was observed for the coupons exposed under simulated WCLL-BB coolant with LiOH (Figs. 1a and 3a), where the average value of the dimension of the crystallites was 1.47 ± 1.09 µm. Conversely, a normal size distribution of the crystallites was observed for the coupons exposed under KOH (Figs. 1b and 3a) and under KOH with 5 ppb Zn (Figs. 1c and 3a) with average dimension of the crystallites equal to 1.23 ± 0.64 µm and 0.98 ± 0.50. Overall, it was observed a refinement of the dimension when KOH and Zn were used in comparison to LiOH as alkalising agent. The darker areas in Fig. 1b are associated with the inner oxide layer, where potential detachments of the outer crystallites occurred during the high-temperature exposure. From the cross-sectional views (Fig. 1d, e, f) and from the statistical violin plots (Fig. 3b and c), the oxide appeared to have a double layer: an inner and uniform oxide layer (below ≈0.4 µm) and an outer oxide layer constituted by the large polyhedral crystallites. It was observed a general refinement of the thickness with a very narrow distribution of the thickness for the coupon exposed to KOH and 5 ppb Zn (Fig. 3b).

The top view of the oxidised EUROFER-97 coupons with the P320 surface simulating a machining process are shown in Fig. 2. Similarly to the OPS coupons, the P320 surface was covered with a uniform oxide layer (Fig. 2a, b and c). For this surface condition, the coupons exposed under LiOH (Figs. 2a and 3a) and KOH with 5 ppb of Zn (Figs. 2c and 3a) presented a bimodal distribution of the crystallites with average value equal to 1.54 ± 0.98 µm and 1.85 ± 1.61 µm, whereas only the coupons exposed under KOH presented a normal size distribution with average value around 0.95 ± 0.55 µm. Similarly, the cross-sectional views of the P320 surface (Fig. 2d, e and f) show a duplex oxide composed of a uniform inner oxide and a coarse outer oxide. From the FIB cross-section analysis, it was observed that the thickness of the outer crystallites (Fig. 3b) and of the inner oxide was decreased especially for the coupons exposed to KOH with 5 ppb of Zn (Fig. 3c) compared to the LiOH only (Fig. 3c). It is also worth mentioning that underneath the inner oxide, an ultrafine-grained layer with a thickness in the range of 0.5–1 µm was observed. It is well known that industrial machining and grinding processes can introduce on steels and Ni-base alloys a locally plastically deformed region formed of ultrafine grains in the range of ≈10 – ≈200 nm^34–39^, named “ultrafine-grained layer”. Finally, it is important to remind that the outer oxide that was observed on the EUROFER-97 coupons is formed by reprecipitation of the cation (e.g., Fe^2+^, Zn^2+^) from the media onto the surface and by comparison with SSs under PWR environment, the outer oxide is not protective^40^. Therefore the oxidation behaviour of the EUROFER-97 cannot be evaluated from the assessment only of the outer oxide, differently from them inner oxide that is generally formed by solid state diffusion and it is more protective^41^.

The dimension of the crystallites (from top surface analysis) and the thickness of the duplex oxide (from FIB cross-sections) was calculated from 150 measurements over ≈4500 µm^2^ and from 60 measurements over a profile of ≈60 µm, respectively per coupon. The summary of the dimensions associated with the outer oxide crystallites and the inner and outer oxide thicknesses, observed on the OPS (blue left violins) and P320 (orange right violins) prepared surfaces, are listed in Table 1 and in the violin plots of Fig. 3. The violin plots show the population (coloured area), the mean value and the top and bottom extreme of the ranges analysed (Fig. 3). Specifically, the plot a of the Fig. 3 shows the distribution of the dimension of the outer crystallites. The plots b and c of Fig. 3 shows the thickness of the outer crystallites (e.g., outer oxide) and inner oxide, respectively, for the EUROFER-97 coupons prepared with the OPS (blue violins) and P320 (orange violins) surfaces. It is important to note that the mismatch between the width (Fig. 3a) and thickness (Fig. 3b) of the crystallites can be associated with the impossibility of exactly stopping the fibbing process exactly at the maximum height of the crystallites and due to the effect of the pressure that might have hindered the vertical development of the crystallites.

**Table 1:** Summary of the size distribution and thicknesses of the oxide formed on the EUROFER-97 coupons that were OPS polished and P320 ground prior to exposure to the different proposed WCLL-BB water chemistries

Water chemistry *pH*_300°C_ = 7.4	Surface condition	Size outer crystallites, µm	Outer crystallites thickness, µm	Inner oxide thickness, µm
LiOH	OPS	1.47 ± 1.09	0.84 ± 0.46	0.38 ± 0.05
	P320	1.54 ± 0.98	1.01 ± 0.48	0.42 ± 0.08
KOH	OPS	0.98 ± 0.50	0.39 ± 0.10	0.32 ± 0.14
	P320	0.95 ± 0.55	0.27 ± 0.17	0.22 ± 0.06
KOH + 5ppb Zn	OPS	1.23 ± 0.64	0.34 ± 0.11	0.32 ± 0.05
	P320	1.85 ± 1.61	0.45 ± 0.37	0.16 ± 0.03

### ATEM microstructural characterisation after high-temperature water exposure

**Fig. 4:**
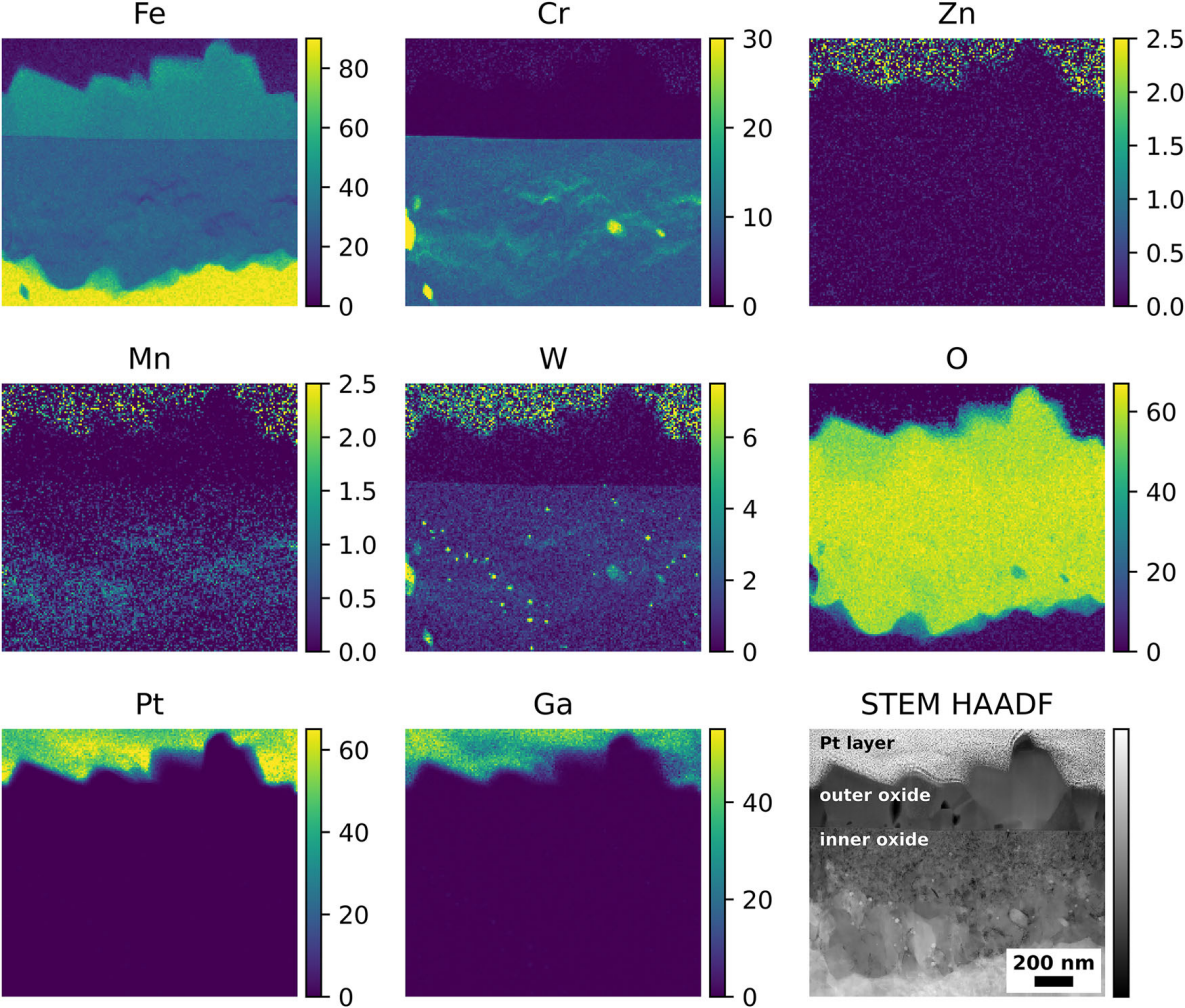
Oxidised EUROFER-97 with an OPS surface after exposure to high-temperature water with LiOH. Atomic % elemental maps extracted from the STEM EDX spectrum imaging dataset and corresponding STEM HAADF image showing the outer and inner oxide layers formed after exposure to high-temperature water with LiOH as alkalising agent.

Figure 4 shows the elemental maps and corresponding STEM high-angle annular dark field (HAADF) image associated with the coupon exposed to water chemistry with LiOH as alkalising agent. The EUROFER-97 with the OPS surface presented a duplex structure composed by an inner polycrystalline layer and an outer oxide with large crystallites. As shown in Fig. 4, the outer oxide layer was Fe-rich, whereas the inner oxide layer contains both Fe and Cr. Moreover, it was observed that the non-oxidised near-surface region microstructure of EUROFER-97 was decorated with randomly distributed Mn and discrete W-rich “spots” associated with M_23_C_6_ carbides^42^. Zn, Ga and Pt elemental maps are shown for clarity and to help the comparisons with the other oxidised EUROFER-97 coupons. The presence of noise in the Zn and W signals in the Pt layer are caused by overlapping peaks between Pt (Pt Lα = 9.435), Ga (Ga Kα = 9.242), Zn (Zn Kα = 8.629) and W (W Lα = 8.140); more precisely, the tails of the strong Pt and Ga peaks overlaps with the weak Zn and W peaks.

**Fig. 5:**
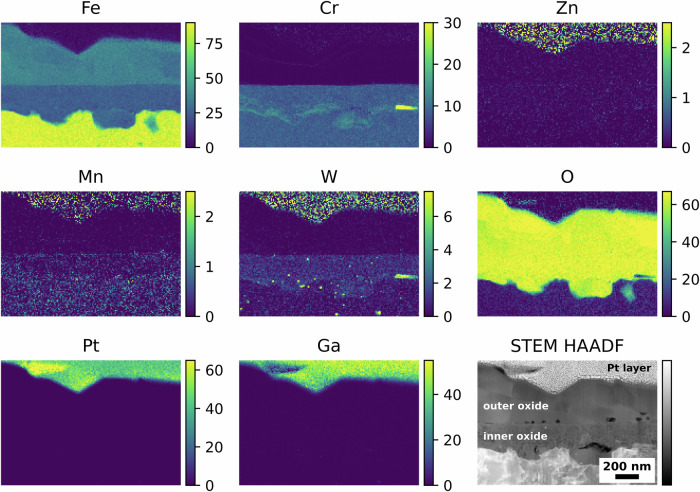
Oxidised EUROFER-97 with an OPS surface after exposure to high-temperature water with KOH. Atomic % elemental maps extracted from the STEM EDX spectrum imaging dataset and corresponding STEM HAADF image showing the outer and inner oxide layers formed after exposure to high-temperature water with KOH as alkalising agent.

Similar results were obtained for the EUROFER-97 coupon that was OPS polished prior to oxidation and exposed to KOH water chemistry, where the outer oxide was Fe-rich and the inner oxide was composed of Fe and Cr (Fig. 5).

**Fig. 6:**
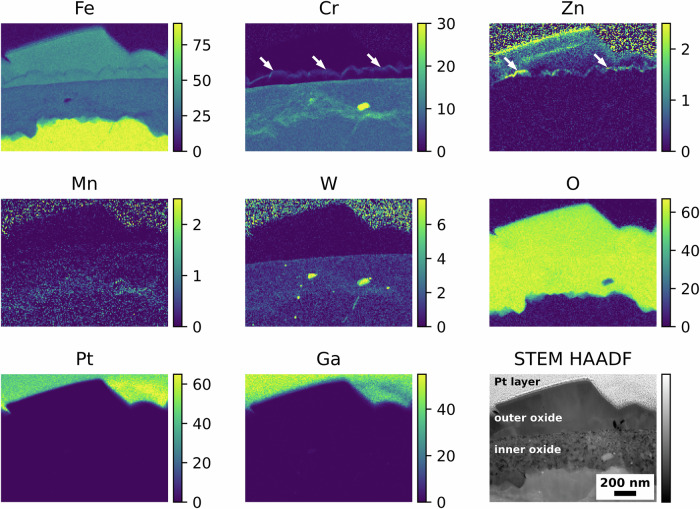
Oxidised EUROFER-97 with an OPS surface after exposure to high-temperature water with KOH and Zn. Atomic % elemental maps extracted from the STEM EDX spectrum imaging dataset and corresponding STEM HAADF image showing the outer and inner oxide layers formed after exposure to high-temperature water with KOH as alkalising agent and 5 ppb Zn. The white arrows in the Cr Kα and Zn Kα indicate the local enrichments of Cr and Zn in the outer oxide layer.

On contrary, the oxide formed on the EUROFER-97 coupon that was OPS polished and exposed to water chemistry with KOH and 5 ppb of Zn (Fig. 6) showed a slightly different behaviour compared to the coupons exposed to the water chemistries without Zn. As expected, also this coupon presented the duplex oxide structure with the outer oxide rich in Fe (Fig. 6) and the inner oxide composed of Fe and Cr (Fig. 6). However, the outer oxide was slightly locally enriched in both Cr and Zn, as indicated by the white arrows in the Cr Kα and Zn Kα maps in Fig. 6, more specifically, Zn was identified both on top of the largest outer crystallites (Zn maps in Fig. 6) and on the top of the smaller crystallites, in the correspondence of a Cr-rich layer (white arrows in Cr and Zn maps in Fig. 6). No Zn was observed in the inner oxide layer, unlike in SS coupons exposed under similar water conditions^42^.

**Fig. 7:**
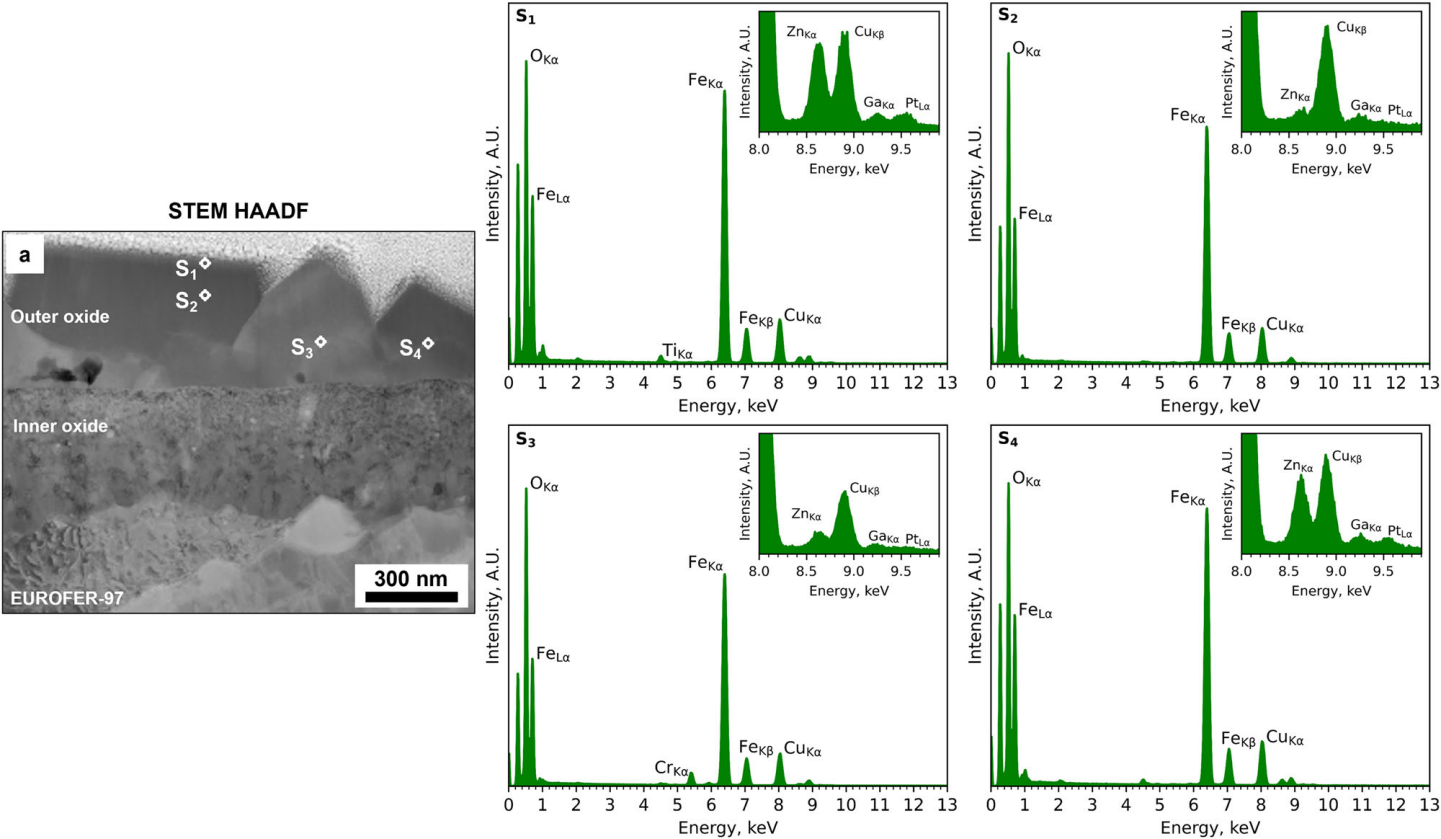
Local STEM EDX analysis of the outer oxide layer formed after exposure to high-temperature water with KOH and Zn. STEM HAADF image and corresponding (S_1_, S_2_, S_3_ and S_4_) STEM EDX point “spot” analyses to confirm the presence of Zn in the outer oxide layer formed on the EUROFER-97 that was OPS polished and exposed with KOH and 5 ppb Zn.

Complementary STEM EDX discrete spot analyses were performed to accurately identify the presence of Zn and Cr from the EDX spectra (Fig. 7) and to eliminate any potential interference from the Pt (Pt Kα = 9.435) and Ga (Ga Kα = 9.242) peaks. A total of four spectra were acquired, and their location is labelled in the STEM HAADF image in Fig. 7. Overall, the spectra (S_1_ – S_4_) solely showed the presence of Fe (Fe Lα at 0.705 keV and Kα at 6.390 keV) and O (O Kα at 0.525 keV), consistent with the presence of Fe_3_O_4_ in the outer oxide. Zn (Zn Kα at 8.639 keV) was observed from the spectrum S_1_, positioned close to the top portion of the largest crystallites, and from the spectra S_3_ and S_4_, positioned on the top of the smaller crystallites. In this latter case, Cr (Cr Kα at 5.415 keV) was also observed and confirmed in correspondence with the Zn-rich layer, thus suggesting the presence of ZnCr_2_O_4_ spinel oxide. No Zn (Zn Kα at 8.639 keV) was detected in the central region of the outer oxide layer (spectrum S_2_). Smaller peaks in the spectra from S_1_ to S_4_ are associated with the presence of Cu (Cu Lα at 0.929 keV), Ga (Ga Kα at 1.098 keV), P (P Kα at 2.014 keV) and Ti (Ti Kα at 4.511 keV), associated with the Cu grid, the FIB milling procedures and redepositions of Ti^2+^ cations from the Ti flow loop and autoclave where the specimens were oxidised.

**Fig. 8:**
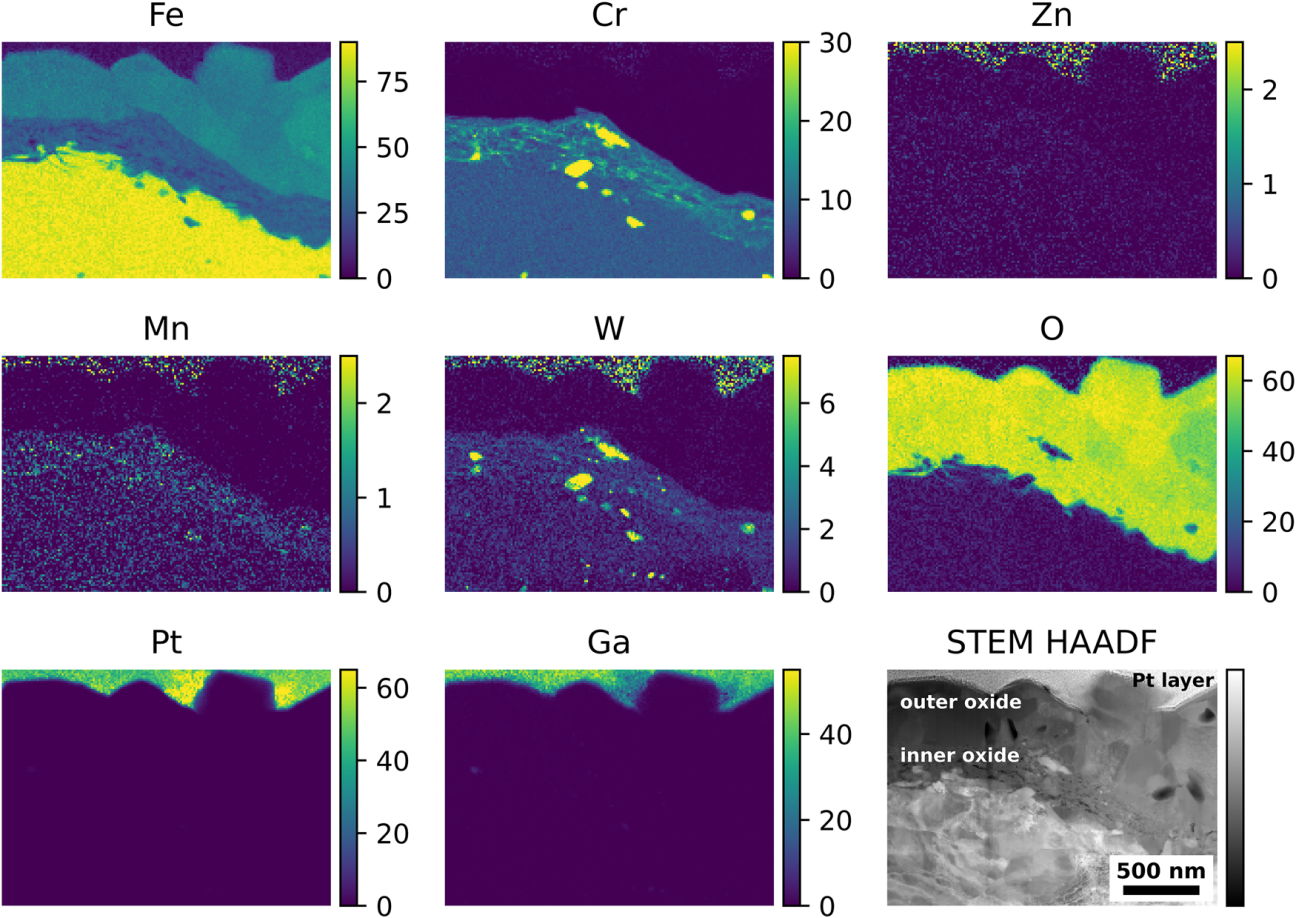
Oxidised EUROFER-97 with a P320 surface after exposure to high-temperature water with KOH. Atomic % elemental maps extracted from the STEM EDX spectrum imaging dataset and corresponding STEM HAADF image showing the outer and inner oxide layers formed on the EUROFER-97 coupon prepared with a P320 surface after exposure to high-temperature water with KOH as alkalizing agent.

**Fig. 9:**
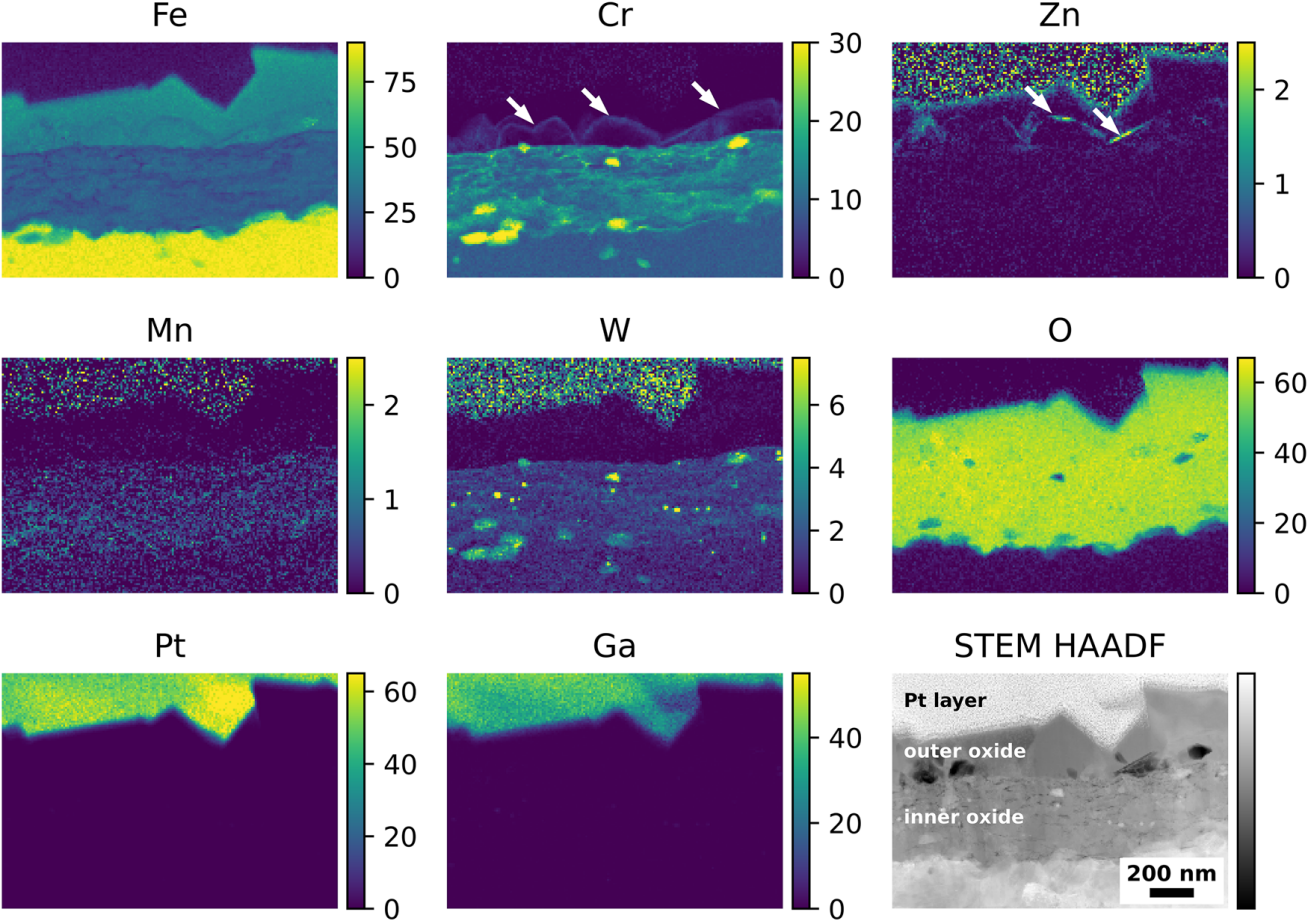
Oxidised EUROFER-97 with a P320 surface after exposure to high-temperature water with KOH and Zn. Atomic % elemental maps extracted from the STEM EDX spectrum imaging dataset and corresponding STEM HAADF image showing the outer and inner oxide layers formed on the EUROFER-97 coupon ground to P320 and exposed to high temperature water with KOH as alkalizing agent and 5 ppb Zn. Please note that the white arrows in the Cr Kα and Zn Kα indicate the local enrichments of Cr and Zn in the outer oxide layer.

Further characterisations were performed on the electron transparent coupons extracted from the EUROFER-97 sample ground to P320 exposed to the various water chemistries. Also in this case, a duplex oxide was observed for both coupons exposed to either KOH (Fig. 8) or KOH with 5 ppb of Zn (Fig. 9). Similarly to the OPS polished coupons, the outer oxide layer of the P320 ground coupon exposed to KOH (Fig. 8) was Fe-rich, whereas the inner oxide layer was rich in both Fe and Cr. It is worth noting the presence of a slightly more localised enrichment of Cr in the inner oxide layer, compared to the EUROFER-97 coupons prepared with an OPS surface; this may be associated with the formation of short-circuit paths induced by the plastically deformed ultrafine-grained layer that promoted the outward Cr diffusion^43,44^. Moreover, also for these coupons it is possible to note the presence of Mn (Fig. 8) in the near surface region, and the presence of W-rich M_23_C_6_ carbides (Fig. 8)^42^, as previously observed for the EUROFER-97 with an OPS-prepared surface (from Figs. 4 to 6).

Zn (Zn Kα in Fig. 9) was detected on the top portion of the large and small polyhedral crystallites forming the outer oxide on the EUROFER-97 coupons that were P320 ground prior to exposure to KOH with 5 ppb of Zn, similarly to what was observed for the EUROFER-97 coupons that were OPS polished prior to exposure to the equivalent environmental condition (Fig. 9). Also, in this case, the Zn was associated with discrete Cr-rich areas, as indicated by the white arrows in the Zn Kα and Cr Kα maps in Fig. 9. The inner oxide was not affected by the presence of Zn in the water chemistry, and it presented similar features as the coupon exposed to KOH water chemistry only (Fig. 8): the inner oxide presented more localised Cr-rich areas, possibly associated with the effect of the ultrafine-grained layer induced by the grinding process^34–38^ and the presence of Mn (Mn Kα in Fig. 9) and W (W Mα in Fig. 9) in the near surface region. It is also worth noting that the presence of W-Cr-rich carbides are not oxidised. A similar oxidation behaviour was also observed for the specimen exposed to LiOH water chemistry and for brevity the associated STEM EDX analyses are not shown in this work.

**Fig. 10:**
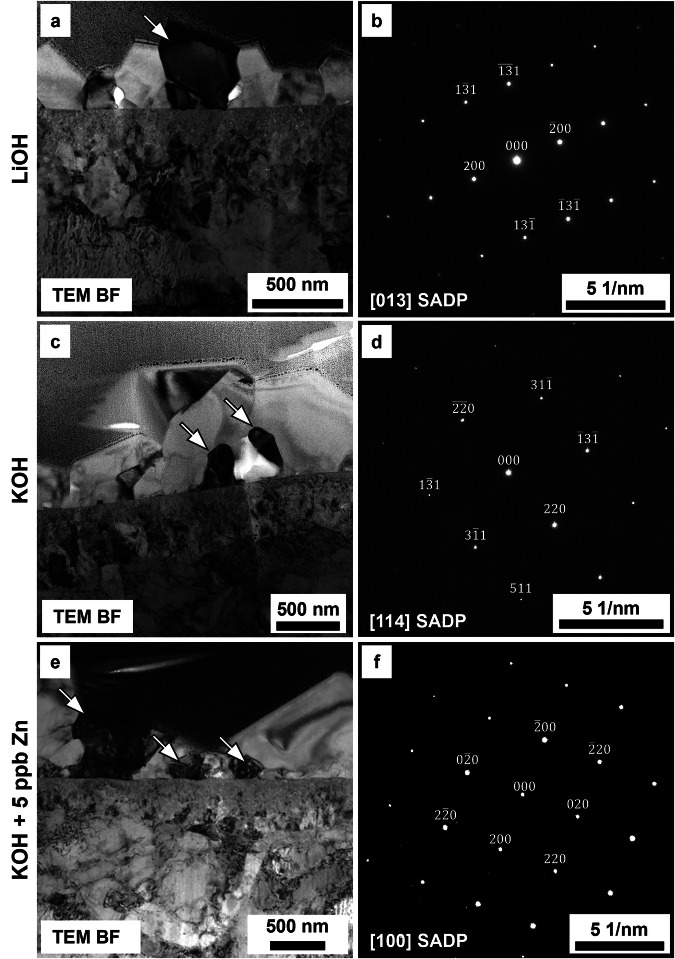
Conventional TEM analyses of the outer oxide layer. **a**, **c**, **e** TEM BF images showing the oxidised surface outer oxide layer for the OPS polished EUROFER-97 coupons exposed to (**a**) LiOH, (**c**) KOH and (**e**) KOH with 5 ppb Zn. The (**b, d, f**) SADPs are associated with the darkly imaging grains (white arrows) of the outer oxide layer for the coupon exposed to (**b**) LiOH, (**d**) KOH and (**f**) KOH with 5 ppb Zn. The diffraction spots are consistent with the presence of Fe_3_O_4_ as outer oxide layer.

Further conventional TEM analyses were performed on the oxide to identify the oxide species formed in the outer and inner oxide layers. The TEM bright field (BF) images in Fig. 10 show the near surface region of the EUROFER-97 sample that was OPS polished exposed to the simulated WCLL-BB water with LiOH (Fig. 10a), KOH (Fig. 10c) or KOH with 5 ppb of Zn (Fig. 10e). The outer oxide layer was identified by indexing the selected area diffraction patterns (SADPs) in Fig. 10b, d and f. The SADPs are associated with the darkly high diffracted grain(s) (white arrow) in the TEM BF images in Fig. 10a, c and e and these results, combined with the STEM EDX elemental maps and discrete spot analyses were consistent with the presence of magnetite (Fe_3_O_4_).

**Fig. 11:**
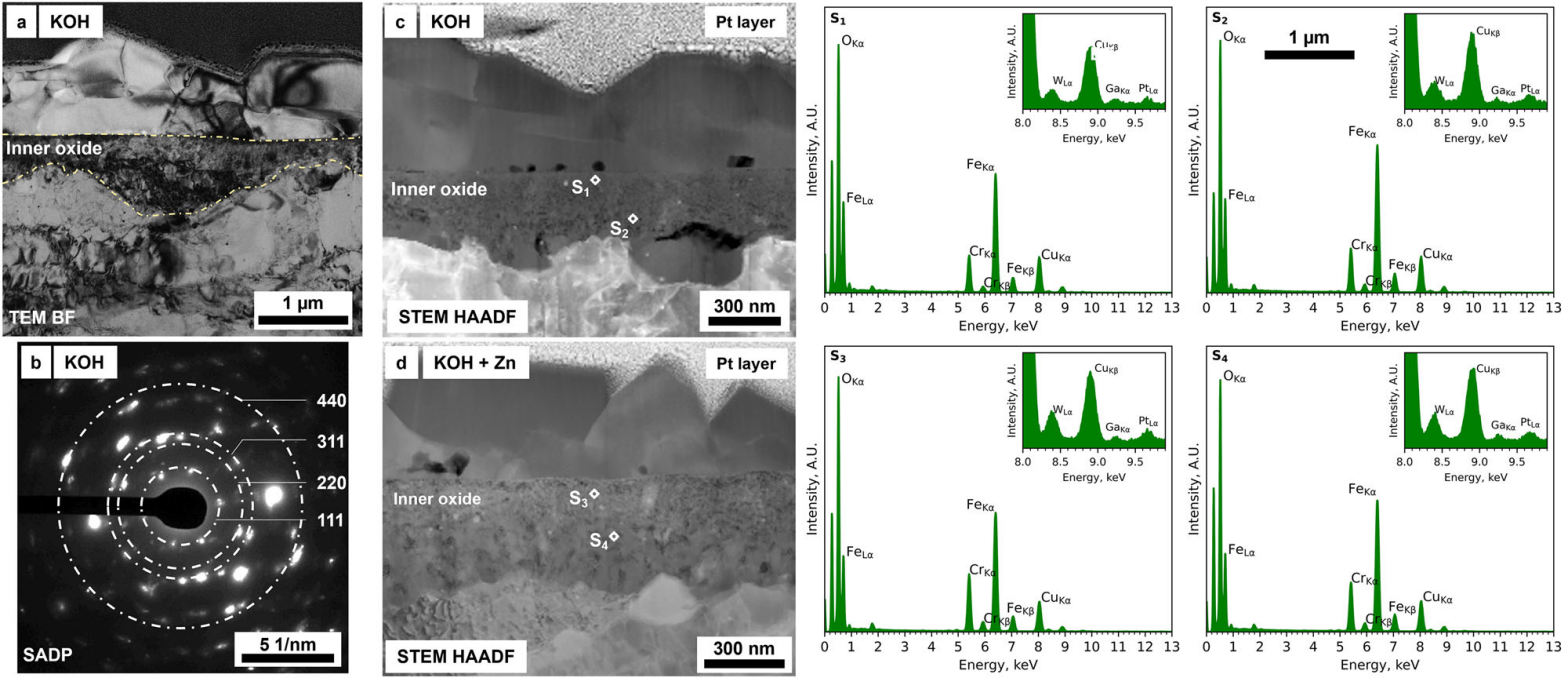
Conventional and analytical TEM analyses of the inner oxide layer. **a** TEM BF image showing the outer oxide layer and the darkly diffracted inner oxide layer (between the yellow lines) on EUROFER-97 polished with OPS and exposed to KOH with the corresponding (**b**) SADP. The discrete diffraction spots associated with the inner oxide layer are consistent with the presence of FeCr_2_O_4_ spinel. **c, d** STEM HAADF images and corresponding (S_1_, S_2_, S_3_, S_4_) discrete “spot” analyses associated with the specimens exposed to (c, S_1_ and S_2_) KOH and (d, S_3_ and S_4_) KOH and 5 ppb Zn. The morphology and elemental composition of the inner oxide layer were observed to be independent from both the alkalising agent and Zn injection.

The TEM BF image (Fig. 11a) shows the inner oxide layer and the associated SADP (Fig. 11a) for the coupon exposed to KOH with *pH*_300°C_ equal to 7.4. The data obtained by indexing the SADP were consistent with the presence of a polycrystalline FeCr_2_O_4_ spinel oxide and found to be independent from the alkalising agent and Zn addiction, as observed also in the other coupons. The conventional TEM data were supported by local STEM EDX “spot” analyses performed in specific locations in the inner oxide layer, showing that the principal elements were Fe (Fe Kα = 6.404 keV and Fe Kβ = 7.058 keV), Cr (Cr Kα = 5.415 keV and Cr Kβ = 5.957) and W (W Lα = 8.398 keV) for the specimens exposed to either KOH (Fig. 11c) and KOH + Zn (Fig. 11d), where W is associated with intergranular carbides^5^. No Zn peak (Zn Kα = 8.629) was observed for the specimen exposed to the Zn-rich water chemistry (inset in spectrum S_4_ of Fig. 11).

## Discussion

Coupons of EUROFER-97 with two different surfaces finishing (OPS *vs*. P320) were exposed for 500 h to 300 °C water with 5 ppb of O_2_ and 44 cc/kg of H_2_ by using LiOH and KOH at *pH*_300 °C_ equal to 7.4 without and with the presence of 5 ppb of Zn. Overall, the Fe_3_O_4_ crystallites forming the outer oxide layer on the EUROFER-97 showed a refinement when KOH and Zn were used, differently from the inner oxide layer that was composed by FeCr_2_O_4_ independently of the water chemistry. Even if the presence of Zn in a concentration under the resolution limit of the STEM EDX “discrete” spot analyses cannot be excluded, it is important to note that this concentration was not sufficient to induce any morphological changes to the oxide. However, no major microstructural differences were observed as a function of the surface conditions (OPS vs. P320). The experimental data were investigated via advanced characterisation techniques and compared to understand the effects of the 1) surface preparation, 2) alkalising agent and 3) Zn injection on the oxide formation for the RAF/M EUROFER-97.

No substantial differences were observed in the oxide layer formed on the EUROFER-97 coupons prepared with either the OPS (Figs. 1, 4 and 5) or the P320 (Figs. 2, 8 and 9) surface: the morphology of the outer Fe_3_O_4_ crystallites was comparable between the OPS (Figs. 1, 4 and 5) and the P320 GRIT (Figs. 2, 8 and 9) prepared EUROFER-97 coupons, thus suggesting very little influence of the machining process on the formation of the outer crystallites. Differences in the oxide morphology were observed, however, in the inner oxide layer: in fact, localised enrichments of Cr (Cr Kα in the elemental maps in Figs. 8 and 9) were observed in the inner oxide layer; this oxide morphology can be explained by the formation of the ultrafine-grained (Fig. 2d, e, f) layer formed after the P320 GRIT process. In fact, the ultrafine-grained layer is formed by nanograins that are locally plastically deformed, thus creating more diffusional and short-circuit paths that can accelerate the outward diffusion of Cr^32,34^. However, despite the presumed enhanced diffusional on ground surfaces^43^, EUROFER-97, which has lower Cr content compared to austenitic stainless steels ( ≈ 9 wt. % *vs*. > 16 wt. %), is less sensitive to surface preparation because it cannot form Cr-rich inner oxide layer. It can be therefore concluded that the oxidation behaviour of EUROFER-97 under this WCLL-BB environmental conditions is not highly influenced by the different surface preparation, differently from what observed in previous studies on austenitic alloys exposed under similar environmental conditions^31–34^.

The *pH*_300°C_ under slightly more alkaline conditions (*pH*_300°C_ = 7.4) was selected to promote the formation of a stable oxide (passivity) and therefore reduce corrosion, especially for EUROFER-97 that presents a lower content of Cr (≈ 9 wt.%) content compared to the austenitic SSs and Ni-base alloys that are widely used in fission. There is no indication that the W/Ta alloying elements have influenced the formation of the inner FeCr_2_O_4_ layer, which is expected to form under nearly all water chemistry conditions in high-temperature water. However, it is also important to note that W, similarly to Mo, has relatively high solubility in high-temperature water. The morphology of the outer crystallites formed on RAF/M EUROFER-97 is comparable with similar ferritic^45^ and SSs^16,46–48^ exposed under similar water chemistries for nuclear applications, however the EUROFER-97 is only covered by Fe_3_O_4_ spinel oxide, differently from SSs that form Fe_3_O_4_ and NiFe_2_O_4_ (nickel ferrite) spinel oxides^28^. The presence of only Fe_3_O_4_ on EUROFER-97 is associated with the fact that this alloy has reduced Ni content^5^ which is specifically selected to avoid transmutation due to the high-energy neutrons expected from the D-T reaction inside the Tokamak reactor. The duplex (inner and outer) oxide formed on the OPS (Fig. 1 and from Figs. 4 to 7) and P320 (Figs. 2, 8 and 9) surface EUROFER-97 coupons presented a similar morphology, with dimension of the crystallites and oxide thickness comparable, however under KOH, the dimension of the outer crystallites was smaller and more homogenous. The reason why the crystallites are smaller in KOH than in LiOH water chemistry is still unclear; however, this phenomenon can be associated with the slightly higher conductivity of water alkalized with KOH compared to LiOH. In fact, it was observed that corrosion deposits under accelerated flow have been shown to slightly decrease when using KOH instead of LiOH as an alkalizing agent^21^. In fact, at the same *pH*, the conductivity of KOH is slightly higher than that of LiOH and allows for more back conduction in the fluid thus decreasing faradaic reactions that are responsible for oxide deposition^49,50^. However, this effect is expected to be negligible in the present study where the stagnant conditions are not associated with electrokinetic deposit^21,51,52^, and therefore further studies are needed to address the refinement effect of KOH on the outer crystallites. However, not much difference can be drawn on the oxidation behaviour as a function of alkalizing agents because the outer oxide is not responsible for protectiveness due to its porous and non-protective nature^32,40^. From the results obtained, EUROFER-97 performs well in high-temperature water, and no detrimental effects of using KOH instead of LiOH were found. Therefore, KOH can potentially be used as an alkalizing agent for the water-cooled section of the breeder blanket component. Overall, EUROFER-97 showed excellent oxidation behaviour under the proposed water chemistry conditions, regardless of LiOH or KOH, thus highlighting its suitability for use in the WCLL-BB components. This is because EUROFER-97 steel formed a duplex oxide layer consisting of a protective inner oxide was rich in Fe and Cr, consistent with the presence of the FeCr_2_O_4_ (iron chromite) spinel, which formed underneath a layer of non-protective large magnetite crystallites.

The addition of Zn to the water chemistry as Zn acetate was investigated to understand its potential effect on the oxide formation and stability on EUROFER-97. The SEM SE (Fig. 1c and Fig. 2c) images and the violin plots (Fig. 3) showed that that overall Zn contributed to the refinement of the outer crystallites, but only for the EUROFER-97 prepared with the OPS surface. In fact, the injection of Zn usually promotes the formation of an outer oxide characterised by a finer crystallite size and an increased number of crystallites^27,28^. However, a bimodal distribution with small (≈ 0.5 µm) and large (≈ 3.7 µm) crystallites were observed for the EUROFER-97 coupon prepared with the P320 surface and exposed to KOH and Zn.

Further STEM EDX and SADP analysis showed that the outer oxide layer was composed of magnetite (Fe_3_O_4_) spinel (from Fig. 4 to Fig. 11), whereas the inner oxide was composed of densely packed Cr-rich oxides consistent with the presence of iron chromite (FeCr_2_O_4_) spinel, as the compositional and diffraction analyses of the inner oxide suggest (from Fig. 4 to Fig. 6 and Fig. 8, Fig. 9 and Fig. 11) in fact Cr is the slowest diffusion elements in steels (Fe^3+^ ≈ Mn^2+^ > Fe^2+^ > Co^2+^ > Ni^2+^ >> Cr^3+^), thus explaining the formation of the outer Fe_3_O_4_ rich layer^53^. Zn and Fe cations are expected to precipitate simultaneously on the surface of the exposed metal (EUROFER-97 in this study), thus forming Fe_3_O_4_ and ZnFe_2_O_4_ (zinc ferrite, franklinite) spinel oxides^54^, however there is a continuous spatial separation of the two species, as Fe_3_O_4_ has an inverse spinel configuration whereas ZnFe_2_O_4_ crystallises in the normal spinel configuration and they are not miscible^55^. However, this mechanism does not completely explain the chemical distribution of the elements in the outer oxide, as Zn (as ZnFe_2_O_4_) was more localised on top of the outer crystallites, and not throughout the entire Fe_3_O_4_ crystallite; further studies at different Zn concentrations are therefore necessary to fully assess the co-precipitation behaviour of Zn and Fe cations, as the current concentration may have not been sufficient to fully saturate the solution and promote the precipitation. The miscibility of the Zn in magnetite is not possible due to a miscibility gap with magnetite (Fe_3_O_4_) in the normal/inverse spinel solvus^27,28,55–57^. In fact, Zn tends to form Zn ferrite (ZnFe_2_O_4_) which is a regular spinel^58^, whereas magnetite (Fe_3_O_4_) is an inverse spinel^59^. It is also worth noting the presence of Cr on top of the smaller outer crystallites (Fig. 6 and Fig. 9); Cr is present in the same location as the Zn-enrichment, thus potentially forming the spinel ZnCr_2_O_4_ (zincochromite). Cr layer formed on top of outer oxides is known to happen under oxygenated water conditions^28^, as Cr oxidises to soluble chromate and reprecipitates on the oxide surface, however the reducing conditions used in this experiment should not induce the Cr enrichment on the top portion of the outer crystallites. Cr-rich oxide can form on SSs under reducing conditions thanks the high stability and lower solubility compared to Ni- and Fe-rich oxides^60–62^, however it is unclear the formation of a Cr-rich oxide on top of the outer crystallites that have been formed by re-precipitation of the Fe cations from the solution, and at this stage it is unclear if this is associated with the water chemistry or with the EUROFER-97 itself as it is not a stainless steel.

The presence of Zn and Cr on the outer surface of the outer crystallites have been possibly caused by local supersaturation conditions established at the oxide/water interface. Also, in this case, zinc ferrite is a normal spinel whereas magnetite is an inverse spinel and therefore this can justify the no incorporation of the two spinel oxides^56^. The EUROFER-97 coupons exposed under 5 ppb Zn water chemistry presented a similar oxide structure to those exposed to the water chemistry without the Zn. However, Zn was only deposited on the top portion of the outer oxide crystallites, in correspondence of a thin Cr-rich layer, thus suggesting the presence of ZnFe_2_O_4_ and ZnCr_2_O_4_ on top of the smaller outer crystallites. No Zn was observed in the inner oxide layer differently from the inner oxide observed in austenitic SSs exposed to similar high-temperature water conditions.

**Fig. 12:**
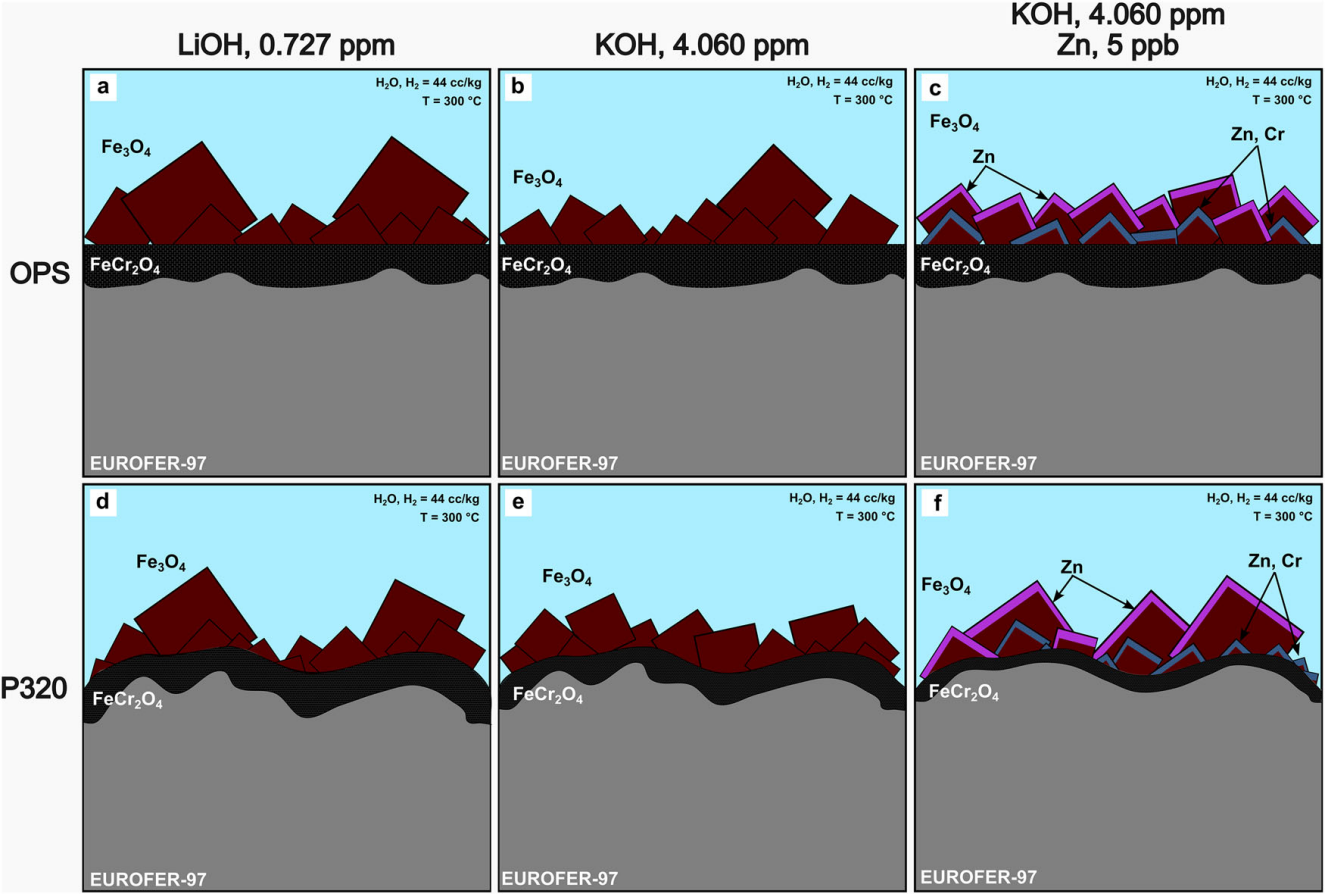
Schematic of the oxide morphologies formed on the EUROFER-97 coupons after exposure to high-temperature water. (**a, d**) LiOH, (**b, e**) KOH and (**c, f**) KOH with Zn for the (**a–c**) OPS and (**d**–**f**) P320 ground prepared coupons. The EUROFER-97 is represented in grey, the inner FeCr_2_O_4_ spinel in black, the outer Fe_3_O_4_ crystallites in marron and the local enrichment of Zn and Zn with Cr in pink and blue, respectively. (For interpretation of the references to colour in this figure legend, the reader is referred to the web version of this article).

A graphical summary of the oxidation processes occurring for the EUROFER-97 with the two different surface conditions (OPS *vs*. P320 ground) and exposed to the different water chemistries (LiOH, KOH and KOH with 5 ppb Zn at *pH*_300°C_ = 7.4) is shown in Fig. 12. The thickness of the inner FeCr_2_O_4_ (black feature in Fig. 12) was independent from the water chemistry for the EUROFER-97 coupons prepared with the OPS surface (Fig. 12a, b and c), whereas a refinement of the outer Fe_3_O_4_ crystallites (maroon crystallites in Fig. 12) was observed under both KOH (Fig. 12b) and KOH with Zn (Fig. 12c). For the coupons exposed under KOH and Zn, the largest and more exposed outer crystallites were decorated with Zn (pink areas in Fig. 12c), whereas the smaller outer crystallites with Zn and Cr (blue areas in Fig. 12c). Differently, the EUROFER-97 coupons prepared with the P320 ground surface presented a slightly different morphology of the duplex oxide: a reduction of the thickness for the inner oxide (black feature in Fig. 12d, e and f) was observed for the coupons exposed to KOH (Fig. 12e) and KOH with Zn (Fig. 12f), whereas the largest crystallites were observed for the coupon exposed to KOH and Zn, differently from the OPS prepared coupon (Fig. 12c). It is plausible that the short-circuit paths induced by the plastically deformed ultrafine-grained layer may have promoted a faster outward diffusion of Fe, thus enhancing the concentration of Fe cations that then reprecipitated onto the surface and forming the large crystallites. Also in this case, the largest more outer crystallites were covered with Zn (pink in Fig. 12f), whereas the other crystallites were covered with both Zn and Cr (blue in Fig. 12f).

## Methods

### Material and specimen preparation

In this study, flat dog-bone SS-J3 type coupons with a thickness of 0.75 mm, a total length of 16 mm and a gauge length of 5 mm were used as flat coupon coupons to study the oxidation behaviour of the RAF/M EUROFER-97 after exposure to simulated WCLL-BB conditions. The coupons were extracted from a EUROFER-97 plate (Heat no. 993394, batch 3) provided by EUROfusion. The EUROFER-97 plate was manufactured by Saarshmiede Freiformschmiede GmbH and hot rolled by Bohler Bleche GmbH & Co. The plate was normalized at 980 °C for 10.8 min and cooled in air, then tempered at 760 °C for 90 min and then finally cooled in air. The chemical composition of the plate is shown in Table 2.

**Table 2:** Chemical composition (wt. %) for the substrate EUROFER-97 used in this study and provided by EUROfusion

C	Si	Mn	P	S	Ni	Cr	Mo	V
0.105	0.024	0.56	0.0025	0.001	0.013	9.08	0.005	0.235
**W**	**Ti**	**Cu**	**Nb**	**Al**	**N**_**2**_	**Ta**	**Co**	**Fe**
1.07	0.001	0.005	0.005	0.009	0.039	0.125	0.007	Bal.

One surface of the SS-J3 coupons was ground with a P320 paper to simulate machining on the surface, whereas the opposite surface was metallographically polished with grinding papers from P320 to P2400, with diamond solution of 3 µm and 1 µm and finally polished with a 24–40 nm SiO_4_ OPS solution. This step was necessary to remove any deformation induced by the previous grinding steps and to obtain a strain-free surface representative of the bulk material^32^. The coupons were finally cleaned with soapy water, rinsed with deionised water, and then dried in a stream of hot air to remove any residue of OPS. Moreover, the effect of surface preparation was investigated to determine if the same impact on oxide evolution^31,33^ and environmentally-assisted cracking^32–34^ behaviour observed in other alloys also applies to the RAF/M EUROFER-97. However, environmentally-assisted cracking for EUROFER-97 is not part of this study.

### Oxidation experiments

The experiments were performed at the Lucideon M + P, 2190 Technology Drive, Schenectady, NY 12308, USA. The flat coupons were exposed to simulated WCLL-BB conditions by using as alkalizing agent LiOH or KOH in different concentrations and with the presence of Zn, as shown in Table 3.

**Table 3:** Water chemistry conditions for mini-tensile coupons exposures for 500 h

Group	Chemistry, ppm	*pH*_300°C_	Conductivity (25 °C), µS/cm	Zn, ppb
1	LiOH, 0.727	7.4	24.4	0
2	KOH, 4.060	7.4	28.1	0
3	KOH, 4.060	7.4	28.1	5

The WCLL-BB chemistry was simulated using a recirculating flow loop connected to a 1-litre titanium autoclave by using high-ultra purity water (≈ 0.055 µS/cm). The deaerated high-ultra purity water was then dosed with either LiOH or KOH and heated up at 300 °C at ≈103 bar (1500 psi) with continuous bubbling of 100% hydrogen at 21 psig over-pressure to achieve ≈4 ppm (44 cc/kg) dissolved hydrogen. The present work aimed to analyse the oxide layer after 500 h of exposure, assuming the oxidation process had reached steady conditions. The environment was selected to be alkaline (*pH*_300°C_ = 7.4 *vs*. a neutral *pH*_*300*°C_ = 5.7) to promote the formation of a stable passive oxide layer and minimise corrosion. This environment is also of interest in commercial power plants, as a similar water chemistry has been proposed in small modular reactors (SMR) currently being developed by Rolls-Royce SMR^63^. The water chemistry was refreshed at an average rate of ~ 3–4 volume exchanges per hour, with control and measurement of ionic and dissolved gas chemistry. The conductivity of the autoclave inlet and outlet water was continuously monitored with two Thornton in-line conductivity meters, and the conductivity values were used to calculate the alkalizing agent (K and Li) and hence monitor the *pH*_300°C_. The water was conditioned to achieve a low oxygen concentration by sparging with nitrogen in the feed tank. After deaeration, 100% hydrogen was continuously bubbled at 21 psig overpressure in the closed loop system (with continuous demineralization). Henry’s law provides a very precise definition of the dissolved hydrogen concentration and has been used in our >40 systems for >40 years, as well as throughout the world in similar laboratories. The actual oxygen level is << 1 ppb, and a Pt reference electrode confirmed that the corrosion potential of the steel was at the expected H_2_/H^+^ potential. Zn was added as an aqueous solution of Zn acetate (Zn(CH_3_CO_2_)_2_) by dilution into the primary flow stream before the high-pressure pump. Autoclave outlet water samples were taken periodically to characterise the Zn concentration. 20 ppb Zn as Zn acetate was used for the first 62 h. After four hours, the outlet Zn concentration was 10 ppb, and after ~58 h the Zn level was 18 ppb. This Zn injection profile was adopted as the coupons and entire autoclave system absorb Zn at higher rates during the initiation exposure than when some Zn is incorporated into the outer parts of the oxide. The Zn inlet was then dropped to 5 ppb Zn, and the water samples thereafter measured a concentration of Zn in the range of 5 – 7 ppb. More details on the experimental system can be found in the references^7,64,65^.

### Material characterisation

A dual beam field emission gun (FEG) – scanning electron microscope (SEM) focussed ion beam (FIB) FEI Helios Nanolab 600i was used to obtain detailed images of the microstructure of the near surface regions after the oxidation tests with a secondary electron (SE) detector with an accelerating voltage of 5 kV and a nominal current of 1.4 nA. For each sample, 5 FIB cross-sections were prepared and analysed using a low voltage (5 kV) to obtain a comprehensive understanding of the cross-sectional overview of the surface oxide formed on the EUROFER-97 after the exposure. In addition, 12 FIB coupons, each containing the oxide film were extracted with the in-situ lift out technique from the oxidised samples, mounted on Cu grid, milled to electron transparency and polished at 2 kV to minimise the ion damage induced by the Ga^+^ ion beam. Transmission electron microscopy (TEM) analyses were performed in FEG-TEM FEI Talos F200X S/TEM with an X-FEG and Super X (4 SDD detectors) for improved scanning transmission electron microscopy (STEM)–EDX spectrum imaging and microanalysis. Analytical TEM was used to characterise the microstructural modification and chemical compositional variation in the region near the surface. The STEM-EDX spectrum image data acquisitions were performed with a dwell time of 200 µs for a total live time of 40 min with a pixel size of 5.978 nm. Complementary point “spot” analyses were performed on the oxide layer for a live-time of 90 seconds. STEM-EDX spectrum image (SI) datasets were acquired with the ThermoFisher Scientific Velox software (version 3.11) and the data were processed as background subtracted and showed as atomic % by using the Python library HyperSpy^66^ and eXSpy^67^. The intensities of the X-rays were extracted using linear curve fitting as implemented in the eXSpy library and the EDX background was modelled using a polynomial of order 6. The presence of noise in the Zn and W signals in the Pt layer of the STEM-EDX elemental maps are caused by the overlapping peaks between Pt (Pt Lα = 9.435), Ga (Ga Kα = 9.242), Zn (Zn Kα = 8.629) and W (W Lα = 8.140), where the tails of the strong Pt and Ga peaks overlaps with the weak Zn and W peaks. The STEM-EDX discrete “spot” analyses were processed with Python library HyperSpy^66^ and plotted with Python libraries Pandas^68,69^ and Matplotlib^70^, as well as the statical violin plots.

## Data Availability

All data generated or analysed during this study are included in this published article.
